# *De novo* transcriptome analysis of *Hevea brasiliensis* tissues by RNA-seq and screening for molecular markers

**DOI:** 10.1186/1471-2164-15-236

**Published:** 2014-03-26

**Authors:** Leonardo Rippel Salgado, Daniela Martins Koop, Daniel Guariz Pinheiro, Ronan Rivallan, Vincent Le Guen, Marisa Fabiana Nicolás, Luiz Gonzaga Paula de Almeida, Viviani Ribeiro Rocha, Milena Magalhães, Alexandra Lehmkuhl Gerber, Antonio Figueira, Júlio Cézar de Mattos Cascardo, AnaTereza Ribeiro de Vasconcelos, Wilson Araújo Silva, Luiz Lehmann Coutinho, Dominique Garcia

**Affiliations:** 1 Departamento de Genética/FMRP/USP, Laboratório de Genética Molecular e Bioinformática, Rua Tenente Catão Roxo, 2501, CEP 14.051- 140 Ribeirão Preto, São Paulo, Brazil; 2 Laboratório de Genômica e Biologia Molecular, Universidade Estadual de Santa Cruz, Rod. Ilhéus-Itabuna BR 415, Km 16, Ilhéus, Bahia CEP 45.662-000, Brazil; 3 CIRAD, BIOS, UMR-AGAP, TA A96/03, Av. Agropolis, 34398 Montpellier, France; 4 Laboratório Nacional de Computação Científica, av. Getulio Vargas, n° 333 Quitandinha, CEP 25.651-075 Petrópolis, Rio de Janeiro, Brazil; 5 Centro de Energia Nuclear na Agricultura/Universidade de São Paulo, Piracicaba, Brazil; 6 ESALQ/USP, Piracicaba, Brazil

**Keywords:** Next generation sequencing, Molecular markers, KASP genotyping chemistry, Rubber tree

## Abstract

**Background:**

The rubber tree, *Hevea brasiliensis*, is a species native to the Brazilian Amazon region and it supplies almost all the world’s natural rubber, a strategic raw material for a variety of products. One of the major challenges for developing rubber tree plantations is adapting the plant to biotic and abiotic stress. Transcriptome analysis is one of the main approaches for identifying the complete set of active genes in a cell or tissue for a specific developmental stage or physiological condition.

**Results:**

Here, we report on the sequencing, assembling, annotation and screening for molecular markers from a pool of *H. brasiliensis* tissues. A total of 17,166 contigs were successfully annotated. Then, 2,191 Single Nucleotide Variation (SNV) and 1.397 Simple Sequence Repeat (SSR) loci were discriminated from the sequences. From 306 putative, mainly non-synonymous SNVs located in CDS sequences, 191 were checked for their ability to characterize 23 *Hevea* genotypes by an allele-specific amplification technology. For 172 (90%), the nucleotide variation at the predicted genomic location was confirmed, thus validating the different steps from sequencing to the *in silico* detection of the SNVs.

**Conclusions:**

This is the first study of the *H. brasiliensis* transcriptome, covering a wide range of tissues and organs, leading to the production of the first developed SNP markers. This process could be amplified to a larger set of *in silico* detected SNVs in expressed genes in order to increase the marker density in available and future genetic maps. The results obtained in this study will contribute to the *H. brasiliensis* genetic breeding program focused on improving of disease resistance and latex yield.

## Background

*Hevea brasiliensis* (*Wild.) Muell.-Arg.* is a tree native to the Brazilian Amazon region and it is botanically classified in the Angiospermae division, class Dicotyledoneae, and family Euphorbiaceae. Many species from the Euphorbiaceae produce latex in specialized cells (laticifers). In the case of *H. brasiliensis*, the latex is a stable emulsion of isoprenoid polymers widely employed to produce natural rubber. In the Amazon, the population of *H. brasiliensis* is estimated to be one of the twenty most abundant tree species [1]. *Hevea brasiliensis* is also the most abundant specie of the genus, with the largest production capacity, accounting for about 99% of all natural rubber produced in the world, and with the greatest genetic variability [2]. Natural rubber is a strategic natural raw material used in more than 40,000 industrial products, including 400 medical devices [3]. Due to its structure and high molecular weight (> 1 million Dalton), natural rubber presents special features such as resilience, elasticity, resistance to abrasion and impact, which cannot be achieved by synthetic polymers [4]. Increased demand for natural rubber on the international market and, consequently, the strengthening of the price, has promoted the rubber cultivation, placing rubber production within the range of highly attractive options available [5].

One of the major challenges for rubber tree cultivation is its adaptation to biotic and abiotic stress. In areas with a notable dry season and low mean temperatures, rubber cultivation is characterized by a long period of immaturity. In tropical regions of Latin America where the high level of relative humidity might be more suitable for rubber development, the climatic conditions are also conducive to the infection of rubber tree leaves by the fungus *Microcyclus ulei*, the causal agent of the South American Leaf Blight. Repeated attacks of this disease cause massive losses of leaves, leading to plant death. The main strategies proposed for avoiding the *M. ulei* damage in plantations involve cultivating genotypes tolerant for dryness and cold in sub-optimal areas and promoting new SALB-resistant and productive cultivars in tropical areas [6]. One of the measures for avoiding *M. ulei* infection takes advantage of the strict high temperature and air humidity conditions for the fungus to reproduce. Based on this requirement, two infection avoidance strategies can be proposed: climatic escape, where leaf exchanges occur during the dry season, when weather conditions are not favorable to fungal sporulation or growing rubber trees in sub-optimal areas (with lower average temperatures and air humidity). Both approaches inhibit *M. ulei* infection, but also may reduce rubber tree yield. The important factor for rubber cultivation is the vegetative fitness of the tree, which is directly reflected in the genetic potential of the cultivated clone [7]. The RRIM600 Oriental clone is classified as susceptible to SALB, and highly productive over a range of temperatures and relative humidity.

Breeding between inbreeds with different characteristics targeting tolerance of biotic and abiotic stress has been identified as an alternative for improving rubber tree growth and production. Overcoming such challenges can be assisted through the development of new strategies and tools in the biotechnology field. Of those tools, we can highlight sequencing of the expressed genome of *H. brasiliensis* (transcriptome), representing the complete set and quantity of transcripts in a cell or tissue for a specific developmental stage and/or physiological condition [8]. In the rubber tree, the identification and characterization of expressed genes may improve our understanding of plant tolerance of biotic and abiotic stress, and the regulation of latex biosynthesis. Thus, the objectives of our study were to capture the transcriptional profile of a large variety of *Hevea brasiliensis* organs and tissues with a view to completing the available reference transcriptomes, then to identify *in silico* SNP and SSR markers and, lastly, develop the first SNPs markers in the rubber tree.

## Results and discussion

### Transcriptome sequencing and assembling

**Figure 1:**
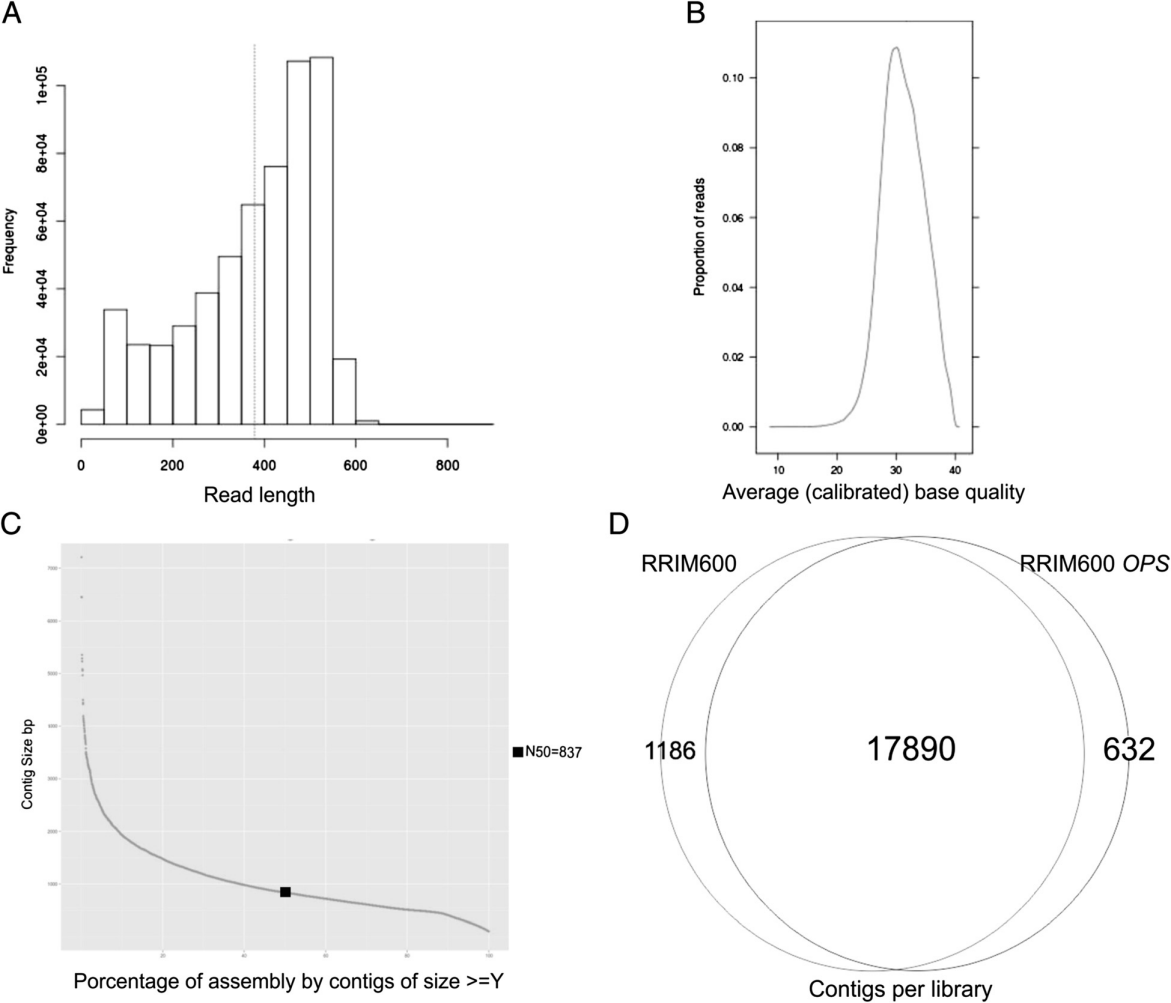
**Run and assembly summary. (A)** Distribution of reads by length, with an average length of 379 bp. **(B)** Overall read quality, lanes with consistently good quality reads have strong peaks to the right of the panel. **(C)** N50 metrics of 837 bp for the assembly. **(D)** Distribution of 19,708 contigs per library.

In order to capture the *H. brasiliensis* mass transcriptional profile with reduced sequencing costs, cDNA libraries were prepared from pooled RNA extracts of different tissues. Total RNA was extracted from 33 organs of the RRIM600 genotype and open-pollinated seedlings of RRIM600 (RRIM600 *OPS* library). Two main cDNA libraries were developed; one from a pool of RRIM600 RNA, and the other from tissues of RRIM600 *OPS* (Table 1). Together these runs produced 525,371 Roche /454 reads of lengths ranging from 40 to 873 bp and a mean of 379 bp (Figure 1A). After checking and trimming for quality scores (Figure 1B), adapters, PolyA/T tails and repetitive elements, 354,949 sequences accounting for 131,895,572 bases were inputted to the NEWBLER 2.7 assembler for contig generation (Table 2). NEWBLER assembly generated 19,708 contigs covering 13,328,059 bases; the N50 metric was 837 bp (Figure 1C) and the mean GC content was 41.50%. On average, each contig received 17.34x coverage ranging from 1 to 3,856 reads. A large number of contigs (90%) resulted from the assembly of reads from the RRIM600 and RIM600 *OPS* libraries (Figure 1D). To overcome the problem of multiple contigs assembled from the same transcript, a scaffolding step by translational mapping (STM) was attempted. The joint assembly predict was built containing 18,867 sequences with an average length of 720 bp ranging from 100 to 10,750 bp. *De novo* transcriptome assemblies may be substantially improved by the addition of a scaffolding step where the contigs belonging to a single transcript are ordered, orientated, and assembled [9]. Approximately 5% of our assembly could be joined onto scaffolds, indicating a low redundancy of contigs. Also, our results of 18,867 scaffolds, N50 = 837 bp, a mean length of contigs equal to 676 bp and other metrics agreed with *de novo* sequencing from other plants [10,11].

**Table 1:** **Organs and tissues used in the RRIM600 and RRIM600 *OPS *** **RNA extracts**

**RRIM600**	RIN*	**RRIM600 *OPS***	**RIN**
Apical meristem	3.0	Cotyledon in the seed (germination stage I)	4.7
Leaflets stage A	3.3	Seedlings (germination stage II)	5.3
Leaflets stage B2	1.8	Stalk (germination stage III)	3.7
Leaflets stage C	2.7	Roots (germination stage III)	3.7
Leaflets stage D	2.5	Seed (germination stage III)	3.3
Petiole (Leaf stage B2)	2.1	Leaves (germination stage IV)	1.6
Petiole (Leaf stage C)	2.7	Stalk (germination stage IV)	3.5
Petiole (Leaflets stage D)	2.7	Roots (germination stage IV)	3.4
Lignified stem	3.0	Seed (germination stage IV)	3.3
Bark (trunk grafted with MDF180 crown)	3.1	Immature seeds with transparent endosperm	2.2
Bark (trunk and crown of RRIM600)	2.8	Immature seeds with white endosperm	1.2
Latex	4.1	Fertilized female flowers	4.5
Raceme	2.9		
Male flowers, mature and immature	3.7		
Columns and wall of fruit lobes	3.3		
Fruit peel	3.4		
Peduncle	2.8		
Seed stage A endosperm	1.2		

Leaf development: Stage A: the preformed leaves in the terminal bud appear; Stage B2: upward leaflets of small size are anthocyanic initially. Then, the lamina reverses and bends to the ground and the anthocyanic color diminishes; Stage C: Rapid lamina growth. The flabby and pale green lamina maintains a drooping position; Stage D: leaves straighten into a horizontal position and become stiff; Seed germination. Stage I: appearance of the radicle; Stage II: appearance of the primary root, side roots and stalk; Stage III: elongation of the primary root, side roots and stalk; Stage IV: elongation of the primary root, side roots and growth of the first two branches. *The RIN software algorithm allows classification of total RNA, based on a numbering system from 1 to 10, with 1 being the most degraded profile and 10 being the most intact.

**Table 2:** **Summary of *Hevea brasiliensis *** **transcriptome sequencing**

**Sample**	**Number of reads**	**Aligned reads**	**Number of contigs**	**Average contig size**	**N50**	Q40 plus*
RRIM600 and RRIM600 *OPS*	523,371	80.69%	19,708	936	969	94.07%

* indicates the bases with a Phred-like consensus quality score of at least 40 (meaning a 1:10000 chance of the base being wrong).

**Figure 2:**
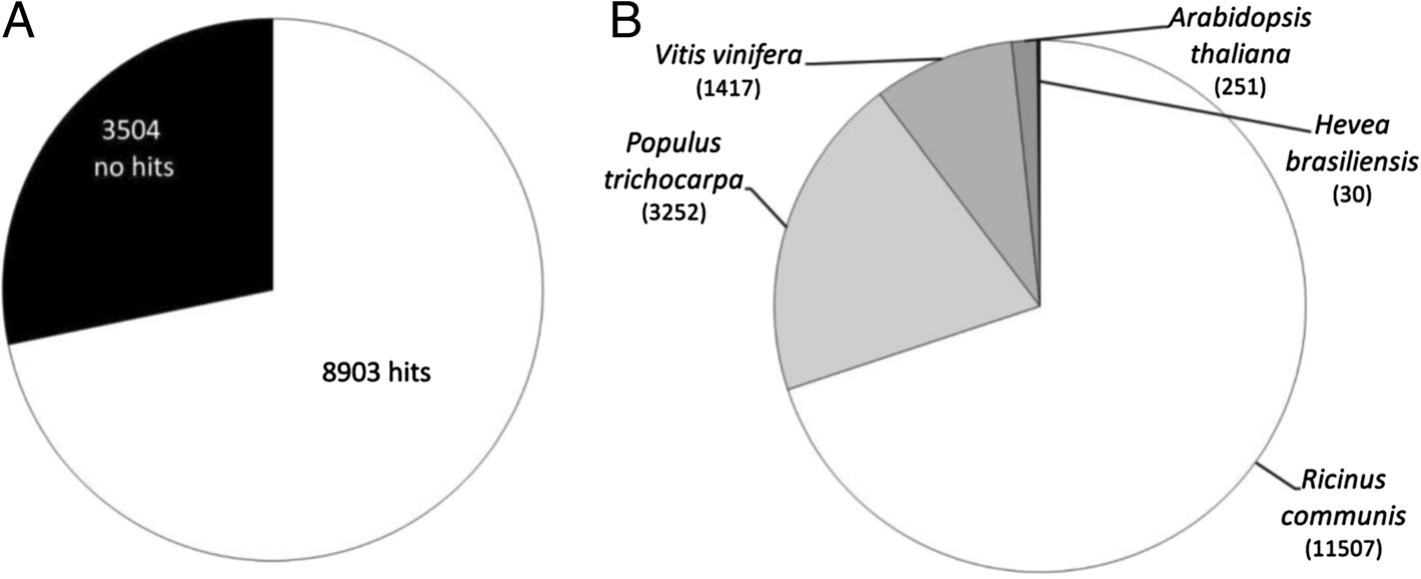
**Comparison by BLASTx with 12,365 *H brasiliensis *sequences deposited in NCBI gene bank. (A)** Matches; **(B)** Distribution for the top hits species.

To verify the consistency of the assembly, a tBLASTx analysis was performed against the existing 9,860 EST sequences from *H. brasiliensis* deposited at the NCBI database by Chow, 2007 [12] generated by Sanger sequencing technology. The results indicated a coverage of 72% of the previously deposited *Hevea* ESTs available in NCBI (Figure 2A). Only 7% presented less than 90% similarity and only 4% had an E-value higher than 1e-40. The high similarity between the assembly and the deposited *Hevea* EST sequences suggested a consistent assembly and good coverage of the *Hevea* transcriptional landscape from all the tissues.

Triwitayakorn et al. [13] sequenced the expressed genome of the *H. brasiliensis* from the vegetative shoot apex of the rubber tree using the 454 platform, obtaining a larger number of reads (2,311,497 reads vs. 525,371 reads obtained here) with a lower average read length (294 vs. 379 bp) but with a similar number and size of the assembled sequences in 19,152 isogroups (theoretically, each one represents a single gene and its variations) ranging between 500 and 1000 bp, while our assembly produced 19,708 contigs with 676 bp on average. Apart from the higher number of reads and the sampling of a few exclusive rubber tree tissues by Triwitayakorn et al. [13], the overall assembly process was very similar.

### Transcriptome Annotation

Contigs were first annotated against a set of plant reference proteins (399,458 entries) by BLASTx. A total of 16,797 contigs identified matches with an E-value cut-off less than 1e-05 (Table 3). In an attempt to obtain a maximum of annotated contigs, a tBLASTx was carried out using the Plant Unique Transcript (PUT) database for *Hevea brasiliensis* ESTs with 4,896 entries in order to cover the transcripts that might lack protein annotation and could not be found in other databases. Subsequently, we performed a BLASTx against the Non-Redundant NCBI protein database with the still unannotated contigs. In total, 2,911 non-annotated contigs were used as input and 299 contigs displayed a high score and a low e-value in the alignment with the *Hevea* PUT database (Table 3). The numbers presented by our Score (median 249.0), Identity (median 85%) and E-Value statistics (median 1-e71) demonstrated a well performed assembly process since few non-matches (~12%) were observed at the end of entire BLAST annotation process. Our percentage of annotated sequences (88%) was quite similar to the transcriptome of Xia et al. [14] who obtained 76% of annotated sequences, of which 65.5% matched with *Ricinus communis* (*vs.* 68% in this transcriptome) (Figure 2B). Differently, for the *Hevea* trancriptome of Triwitayakorn et al. [13], 48% of sequences matched with *Manihot esculenta*, followed by *R. communis* (45%).

**Table 3:** **Summary of *Hevea brasiliensis *** **sequential annotation**

**Database**	**Annotated sequences**
Plant RefSeq protein	16,797
*H. brasiliensis* PUT	299
NR NCBI protein	70
GO	8,725
COG	15,172
KEGG	5,059
Not annotated	2,542

**Figure 3:**
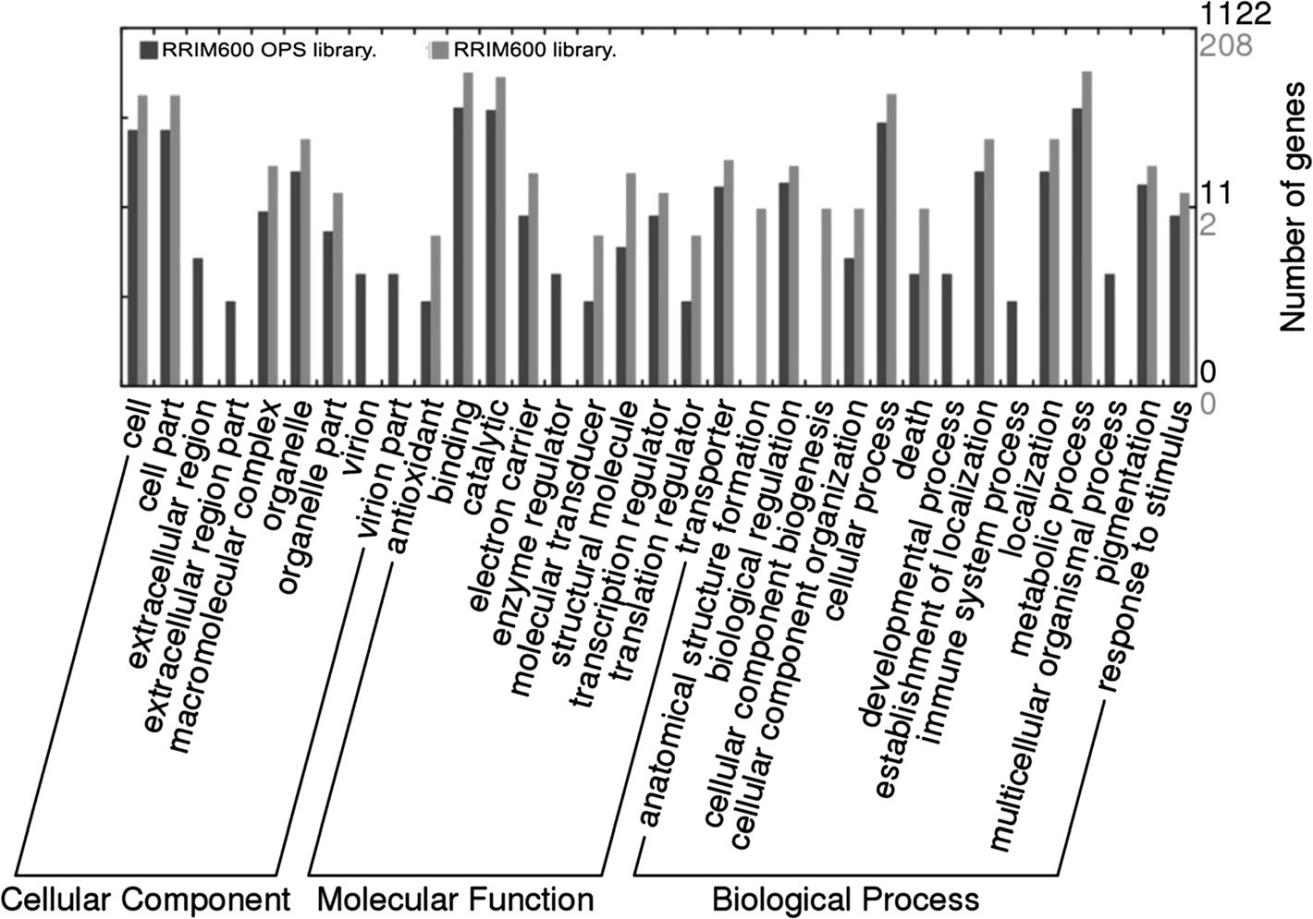
**Summary of functional analysis by GO terms between the RRIM600 library and the RRIM600 *OPS *** **library divided into three main categories, “biological process”, “cellular component” and “molecular function”.**

**Figure 4:**
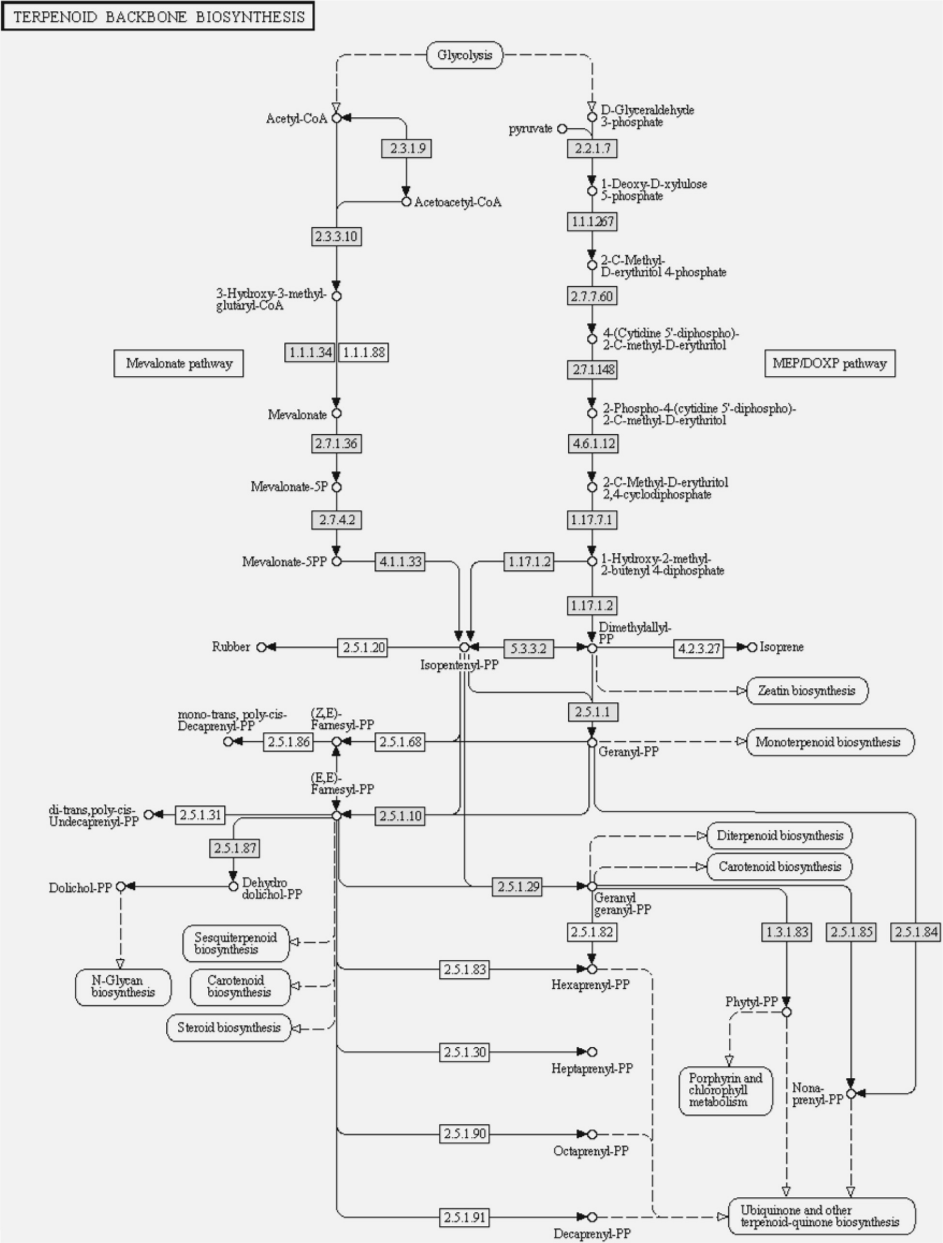
**Transcriptome sequencing successfully identified most of the genes of the main active pathways.** The terpenoid biosynthesis pathway where gray boxes indicate the identified genes in our libraries.

**Figure 5:**
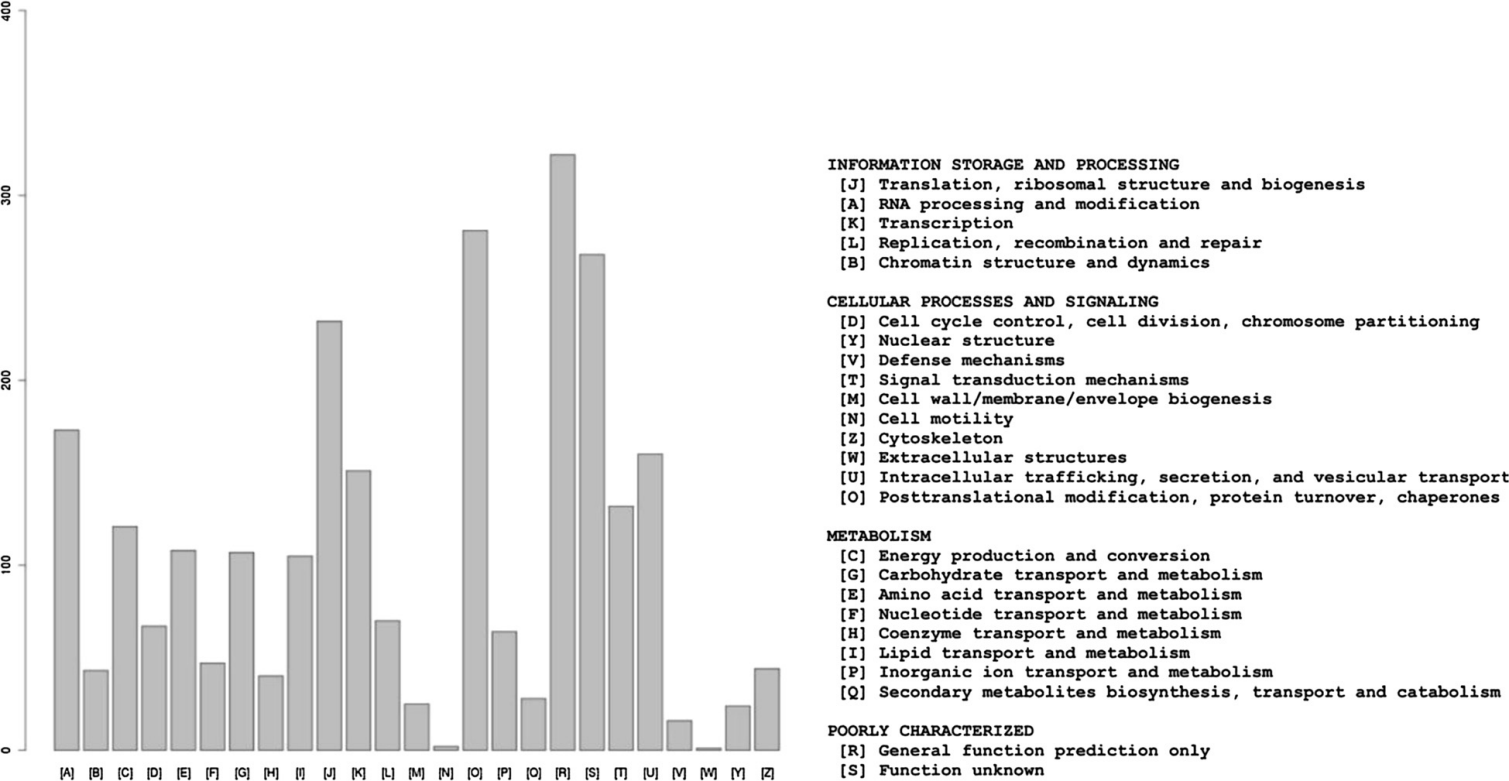
**Cluster of orthologous classification.** 76% of all sequences were successfully annotated under 25 clusters and 2,631 groups. The orthology cluster described as ‘unknown function’ and general ‘prediction only’ accounted for 22% of annotations; another major clustering was related to translational and post translational functions (19%).

To classify contigs correctly, a search for protein signatures was performed by InterPro scan on Open Reading Frames (ORFs) from each contig for Protein Domains, PANTHER (Protein Analysis Through Evolutionary Relationships) evidence, TIGR [Hidden Markov Models (HMMs) for protein sequence classification, and associated information] and Fingerprint (group of conserved motifs used to characterize a protein family) evidence, resulting in 3,521 IPR signatures; 367 protein family fingerprints; 2,655 protein domain families; 4,970 PANTHER families; 794 different structure families; and 419 TIGR families. These protein domains were associated with 46 GO terms (taking into consideration only those up to the second level of the GO hierarchical tree) belonging to the three GO categories (20 ‘biological processes’, 11 ‘molecular functions’ and 15 ‘cellular components’) (Table 3). The GO terms ‘cell part’, ‘binding activity’, and ‘metabolic processes’ were the most represented classes in each GO category (Figure 3). Also, five genes (represented by 7 contigs) implicated in the mevalonate pathway (MVA) and consequently in natural rubber biosynthesis, were identified (Figure 4). COG analysis successfully classified 15,172 out of 19,708 sequences on 2,631 groups (Table 3). The orthology cluster described as ‘unknown function’ and general ‘prediction only’ accounted for 22% of annotations; another major clustering was related to translational and post translational functions (19%) (Figure 5). The search for genes involved in metabolic pathways resulted in 5,059 contigs annotated in 134 KEGG orthologies (Table 3), distributed in ‘Metabolic Processes’, ‘Enzyme Families’, ‘Genetic Information Processing’, ‘Environmental Information’ ‘Processing’, and ‘Cellular Processes’.

Transcriptome sequencing identified the genes (134) of the main active pathways confirmed by KEGG, covering the majority of enzymes in key processes such as ‘plant hormone signal transduction’ (39/41); ‘plant-pathogen interaction’ (27/50); and ‘photosynthesis’ (46/63) indicating that the effort to capture a global transcriptome landscape was achieved, demonstrated by the diversity of KEGG and GO annotation. Xia et al. (2011) [14] obtained 125 KEGG pathways, mainly distributed on ’metabolic pathways’, ‘spliceosome’, and ‘plant-pathogen interaction’, while our most enriched pathways where ‘Ribosome’, ‘Spliceosome’, and ‘RNA transport’.

### Comparisons with *Hevea* EST resources

**Figure 6:**
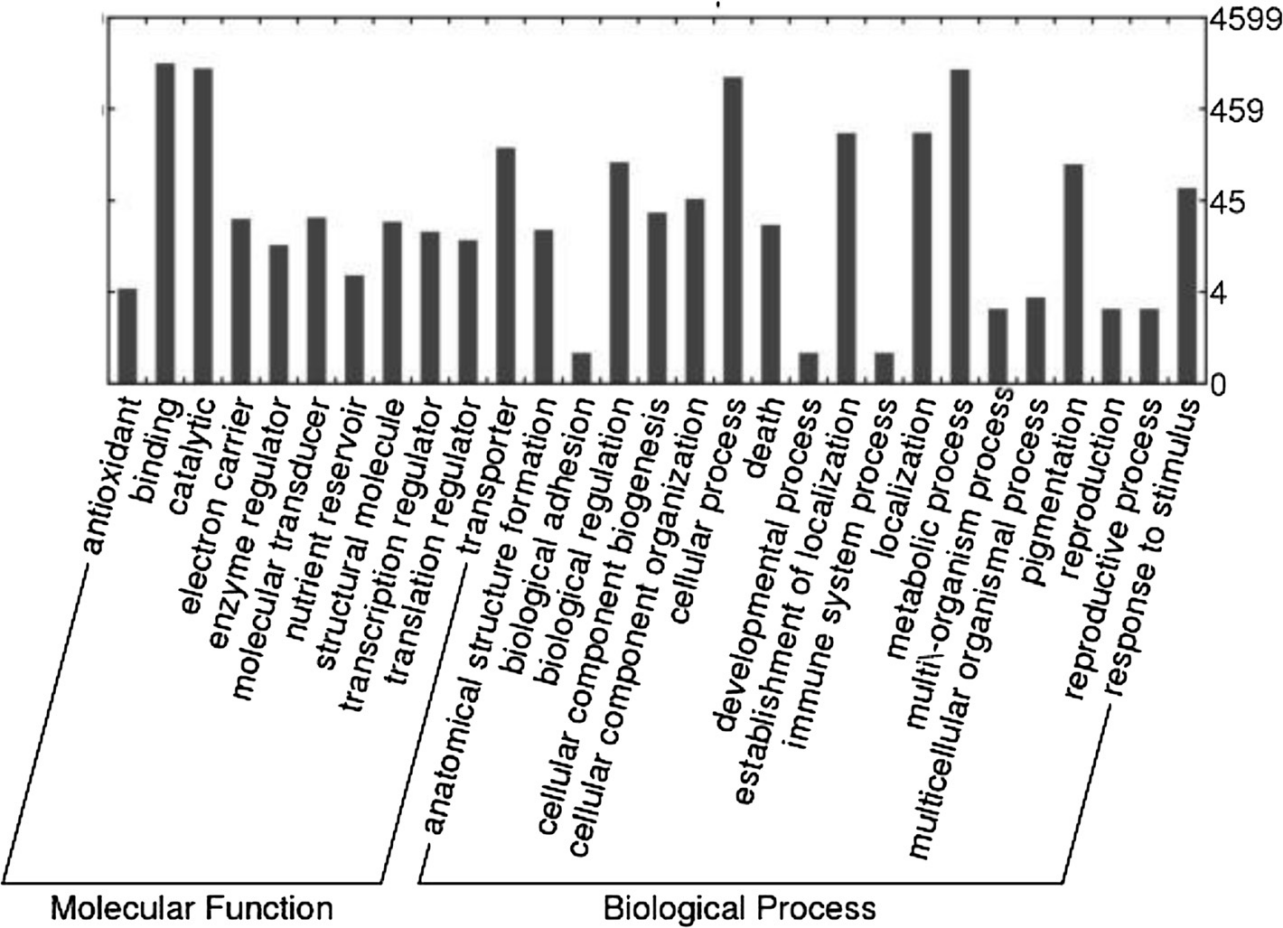
GO terms histogram associated with “biological process” and “molecular function” for the 813 unique terms for the 8,792 novel genes.

To verify the amount of new information provided by our assay, a tBLASTx was performed against the existing 39,034 EST sequences deposited in the NCBI public database related to *Hevea brasiliensis* (October, 2013)*.* Of the 19,708 sequences, almost half of them (8,792) found no hits with an E-value cut-off of 10^-5^. Of these 1,164 were successfully annotated in the KEGG database, on 647 different KEGG orthologies, of which 356 were annotated as different enzymes belonging to metabolic pathways. Also, out of the 8,792 sequences, 3,949 were successfully annotated by searches for protein signatures on 2,095 different Interpro terms and 813 uniq GO terms (Figure 6). When ranked by uniqueness (1- average semantic similarity of a term with all other terms; more uniq terms tended to be less dispensable) [15] the main biological process was the “mannose metabolic process”, for cellular components it was the “cis-Golgi network” and for molecular functions “ammonium transmembrane transporter activity”.

To gain an understanding of our transcript sets, comparing our contig set (19,708 sequences) with the read set originated from Triwitayakorn et al. [13], we identified 2,833 sequences with no correspondence, indicating new gene contributions from our libraries. Of those, 367 were exclusive sequences from the RRIM600 library and 564 originated from young tissues (RRIM600 *OPS* library). The 2,833 novel sequences were annotated on 56 GO terms in three ontologies, being ‘cell part’ as the most representative on the ‘Cellular Component’ term, ‘Metabolic Process’ for the ‘Biological Process’ term, and ’binding’, for ‘Molecular function’. Of the 2,833 new sequences identified in this study, 31 sequences were identified as genes related to plant cell dehydration processes. The most frequent was annotated as a Heat-shock proteins (Hsps)/chaperones with 21 occurrences.

### Screening for EST-SSR markers

For many plant species, large numbers of expressed sequence tags (ESTs) have been generated although low numbers of validated EST-SSR and SNP markers are available for plants, especially for non-model plants. For the rubber tree, most of the available marker resources are isozyme, RFLP, AFLP and SSR markers [16]. SSR markers are today mainly obtained by a traditional method of SSR marker development, such as genomic-SSR hybrid screening and selective (or not) amplified microsatellite enrichment [17-21]. Recently, new EST-SSR markers were identified and proposed by several authors from transcriptomic data [13,22-24]. SSRs are typically co-dominant markers, proved to be useful in assessing population structure, determining relationships between closely related species and QTL mapping. Although SSR markers derived from expressed sequences are considered less informative due to DNA sequence conservation in the transcribed region [25], such markers are cost-effective and considered as functional sequences [19].

In our work, out of the 19,708 contigs examined, a total of 1,397 SSRs, formed by 187 different motifs, were identified in 1,148 sequences, with 152 contigs containing more than one SSR locus. The most frequent SSR type found was mononucleotide repeats with an average size of 12.56 bp and 75 bp repeats as the longest (Table 4). Feng et al. [22] searching for EST-SSRs in *Hevea brasiliensis* public databases successfully identified 799 loci on 10,829 EST sequences, one in every 2.25 kb of EST from the rubber tree. Here, we detected a lower SSR/Sequence frequency (1/5.2 kb), but the total number of identified SSR loci seemed to tally between the two studies, whereas the sequence analyzed/SSR discovery ratio was 14.1, similar to the value of 13.5 identified by Yu et al. [19]. The proportion of mono, di and trinucleotide repeats (41%, 20% and 37%) was more balanced in our assay than in the Yu study (45%, 42%, 11%) [19]. Triwitayakorn et al. [13], also found a lower distribution frequency (1/3.3 kb) than Yu et al. [19], but still higher than ours. Discrepancies between studies may come from differences in the methods, and the limitations of 454 technology when dealing with homopolymers.

**Table 4:** Distribution of identified SSRs according to SSR motif types and repeat numbers

**Type of Repeat**	**Number of repeat units**												
	**5**	**6**	**7**	**8**	**9**	**10**	**11**	**12**	**13**	**14**	**15**	**>15**	**Total**
Mononucleotide repeat	0	0	0	0	0	176	115	62	50	27	26	111	567
Dinucleotide repeat	0	110	38	36	22	16	12	7	13	4	2	18	278
Trinucleotide repeat	271	117	45	29	18	16	5	2	5	0	1	1	510
Tetranucleotide repeat	9	1	2	0	0	0	0	0	0	0	0	0	12
≥ Pentanucleotide repeat	22	5	0	2	1	0	0	0	0	0	0	0	30

### Screening for SNV markers

**Figure 7:**
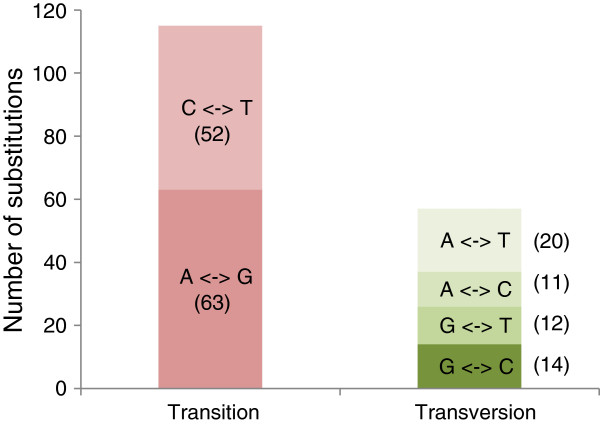
**Classes of single nucleotide polymorphisms detected and validated in 454-derived EST contigs of *Hevea brasiliensis*.** The number of each type of single-base substitutions is indicated in brackets.

Out of the 19,708 sequences obtained in this work, 889 contigs presented single nucleotide variants (SNVs) with more than 4x coverage, an average Q20 quality score, a minimum of 2 supporting reads at a position to call variants, ranging from 1 to 18 substitutions per single contig. These variations accounted for a total of 2,191 predicted biallelic SNVs on a total length of 13.3 Mb of consensus sequences, corresponding to an average of one SNV every 6.0 kb. This density was less than that reported by Pootakham [26] in the rubber tree (1 SNP/1.5 kb), probably due to the greater stringency of our SNV detection parameters. Most of the detected and validated nucleotide variants were transitions (66.8%), with transversions only accounting for 33.0% (Figure 7), which was close to Pootakham’s results [26]. Of those, 260 contigs presented nucleotide variation only on reads derived from the RRIM600 library totalizing 480 predicted SNVs for this particular library, while 362 contigs presented variations from consensus only on reads originating from the RRIM600 *OPS* library in a total of 666 predicted SNVs (Table 5).

**Table 5:** Characterization of the 2,191 identified SNVs associated with 889 contigs

**Nucleotide variation**		**Number of SNVs**	**Nonsense**	**Number of associated contigs**	**SNV position**		
					**Coding sequence**	**UTR**	**Non coding sequence**
Exclusive to the RRIM600 library	Non-synonymous	339	20	161	326	13	0
	Synonymous	141		99	98	27	16
	*Total*	*480*		*260*	*424*	*40*	*16*
Exclusive to the RRIM600 *OPS* library	Non-synonymous	470	20	225	464	6	0
	Synonymous	165		115	90	45	30
	Non-determined*	31		22	-	-	-
	*Total*	*666*		*362*	*554*	*51*	*30*
Common for both libraries	Non-synonymous	900	39	211	804	96	0
	Synonymous	145		56	95	31	19
	*Total*	*1.045*		*267*	*899*	*127*	*19*

* No CDS.

Out of the 2,191 predicted SNVs there were 1,877 substitutions on CDS regions (predicted protein coding sequence) for 889 contigs, which resulted in 1,594 non-synonymous substitutions on 597 contigs. A total of 283 SNVs was observed on non-coding regions. Of all the detected variations, 1,594 were potential non-synonymous substitutions, accounting for 78% of all variations, indicating a high level of genetic variability.

Using a similar strategy, Barbazuk et al. [27] screened maize ESTs from shoot apical meristem by 454 searching for SNPs from two inbred lines and the data were anchored onto the sequence of the maize genome. An initial number of 36,000 putative SNPs was detected after the alignment of nearly 260,000 and 280,000 transcripts of both inbred lines. This figure fell to 7,000 putative SNPs after post-processing. Our strategy was different, using identification parameters with high stringency, allowing the prediction of only 2,191 SNVs, but with an average quality of Q20 and a coverage ≥4x. This strategy proved effective as demonstrated by the validation of 172 out of 191 putative SNPs (90%) using an allele-specific amplification strategy. Similarly, Barbazuk et al. [27] identified variants exclusive to each inbred maize line and polymorphic sites with a different depth by inbred line. The validation of a subset of SNPs by PCR amplification and Sanger sequencing revealed a validation rate over 85%. These data suggest that the computationally identified SNVs represented ‘true’ polymorphisms even for low ESTs-coverage regions, suggesting that 454-based transcriptome sequencing is an excellent method for the high-throughput acquisition of gene-associated SNPs. In the same way, Novaes et al. [28] studying multiple tissues and genotypes of *Eucalyptus grandis*, a non-model plant, on a 454 platform, sequenced and assembled 148 Mbp ESTs from 1,024,251 reads identifying 23,742 SNPs, of which 83% were validated by Sanger sequencing in a sample of 337 SNPs.

From our data, non-synonymous SNVs accounted for 64% (1,211) of overall variations occurring in CDS regions. For non-synonymous changes it is not possible to directly determine how much the amino acid change will affect the protein structure, stability or localization. In Eukaryotes, changes in the protein isoelectric point (iP) may directly influence the localization and reactions of proteins, and it is reasonable to assume that alterations to the global protein iP may interfere in interactions between proteins and complexes. Alendé et al. [29], studying the evolution of iP over mammalian proteins, showed that insertions/deletions were the main reason for the shift in iP and suggested that shifts in iP might be related to the gain in additional activities, such as new interacting partners or preferences for orthologous or isoforms.

From the calculated iP and molecular weight (Mw) for the mutated and non-mutated contigs that displayed non-synonymous mutation, the variation in iP ranged from -1.95 to 1.09, and the Mw from -97.11 kDa to 66.08 kDa. A major alteration in iP was observed on a conserved hypothetical protein from *Ricinus communis* (alteration of -1.95 over original iP) annotated as a *Ricinus communis* conserved hypothetical protein, and 1.09 iP variation over the non-consensus sequence annotated as glycotransferase activity (GO: 0004579), KEEG (K12670) Glycan Biosynthesis. The characterization of mW and iP for the protein sequences translated from contigs with non-synonymous mutation showed that the mW from the mutated sequences was altered, but 250 sequences did not demonstrate any changes in iP values. Flegr [30] suggested that, since cell cytoplasm pH is stratified ranging from 6.4 to 7.2 in Eukaryotes, changes in the protein isoelectric point may directly influence the localization and reactions of proteins, and it is reasonable to assume that alterations to the global protein iP may interfere in interactions between proteins and complexes.

Substitutions outside coding regions (here 247 SNVs) are often linked to gene regulatory regions and may affect events, such as gene splicing, messenger RNA degradation or non-coding RNA sequences, and therefore usually called eSNP/V (expression single nucleotide polymorphism/variant), therefore becoming an interesting feature for biotechnological uses. Here, we were able to identify 2,191 mutations associated with 889 contigs. The results obtained by Barbazuk et al. [27] and Novaes et al. [28] gave sufficient evidence about the reliability of the 454 sequencing platform for SNV identification in transcriptomic data, constituting an important feature for 454 data analysis.

### Analysis of genetic diversity

Out of a set of 191 SNVs detected *in silico*, 172 SNPs were shown to be polymorphic among the 23 tested *Hevea* genotypes belonging to 3 species and resulting from different breeding programs (Table 6). *PIC* ranged between 0.04 and 0.38, and heterozygosity varied from 0.04 to 0.5 (Additional file 1: Table S1). Ten SNPs displayed the same heterozygous combination for all the genotypes and were not included in the analysis of genetic diversity.

**Table 6:** **Genealogy of 23 *Hevea *** **spp. genotypes**

**Genotype**	**Species**	**Genealogy**
F4542	*H. benthamiana*	Primary clone
PUA8	*H. pauciflora*	Primary clone
PA31	*H. pauciflora*	Primary clone
MDF180	*H. brasiliensis*	Primary clone
PFB5	*H. brasiliensis*	Primary clone
Fx2784	*H. brasiliensis*	Unknown
PMB1	*H.brasiliensis*	Primary clone
FDR5788	*H.brasiliensis*	Harbel8 x unknown
RRIC100	*H.brasiliensis*	RRIC52 x PB86
CMB104	*H.brasiliensis*	IRCA109 x PFB5
CMB114	*H.brasiliensis*	IRCA109 x PFB5
IRCA109	*H.brasiliensis*	PB5/51 x RRIM600
PB314	*H.brasiliensis*	RRIM600 x PB235
RRIM600	*H.brasiliensis*	Tjir1 x PB86
PR107	*H.brasiliensis*	Primary clone
PR255	*H.brasiliensis*	Tjir1 x PR107
GT1	*H.brasiliensis*	Primary clone
IRCA130	*H.brasiliensis*	PB5/51 x RRIM600
PB235	*H.brasiliensis*	PB5/51 x PBS/78
PB260	*H.brasiliensis*	PB5/51 x PB49
PB217	*H.brasiliensis*	PB5/51 x PB6/9
Fx3899	*H. benthamiana* x *H.brasiliensis*	F4542 x Avros363
FDR5597	*H.brasiliensis*	Harbel68 x TU42-525

AVROS: Algemene Vereniging Rubberplanters Oostkust Sumatra; CMB: CIRAD Michelin Brazil; F: Ford; FDR: Firestone Dothidella Resistance; Fx: Ford Cross; IRCA: Institut de Recherches sur le Caoutchouc, Ivory Coast; MDF: Madre de Dios Firestone; PA and PUA: *H. pauciflora*; PB: Prang Besar, Malaysia; PMB1: Plantação Michelin da Bahia, Brazil; PFB: Pé Franco Brasileiro; RRIC: Rubber Research Institute Ceylon, Sri Lanka; RRIM: Rubber Research Institute of Malaysia; Tjir: Tjirandji, Indonesia; TU: Turrialba, Costa Rica.

**Figure 8:**
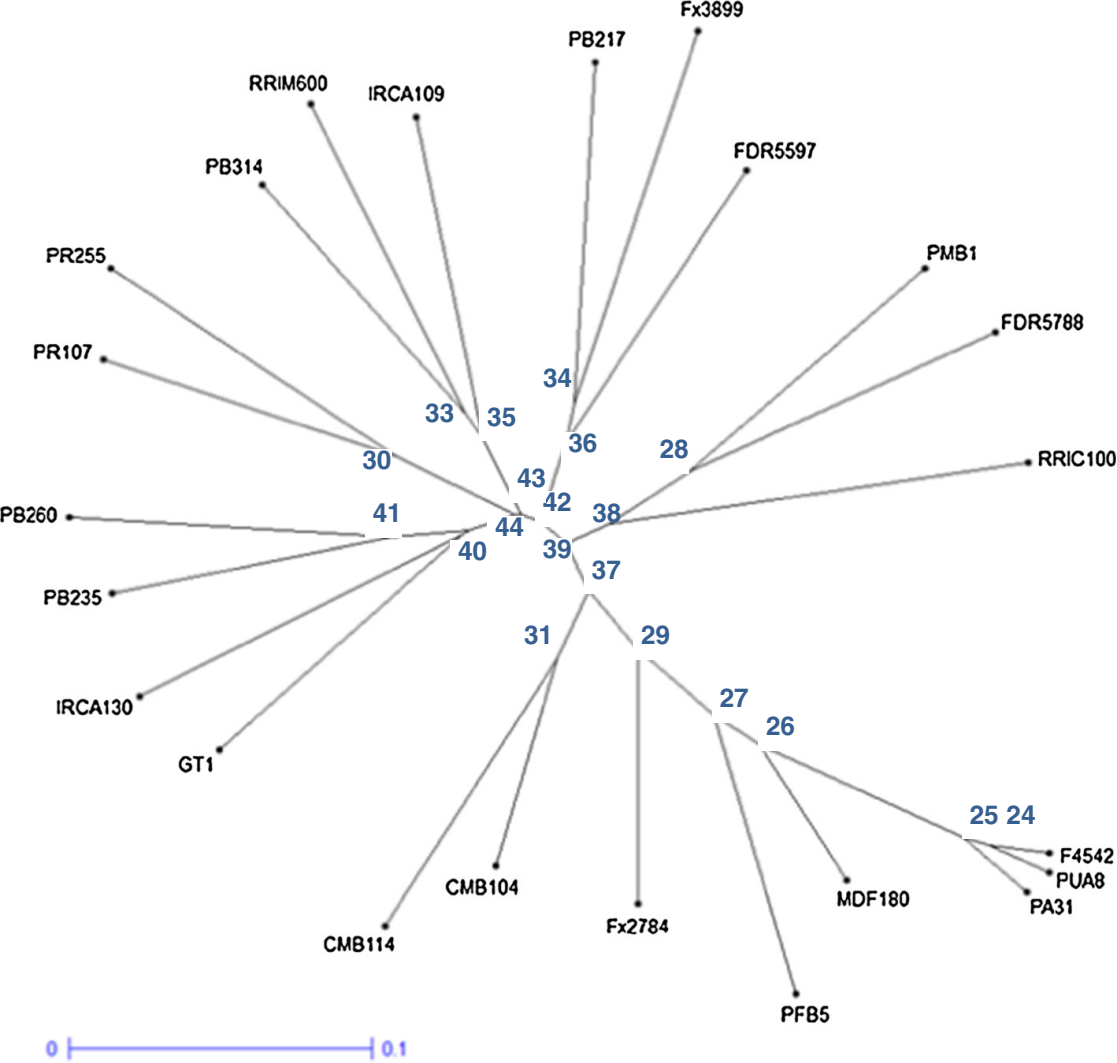
**Neighbor Joining tree illustrating relashionships between 23 *Hevea *spp. genotypes analyzed with 162 SNP loci.** Boostrap values (1000 replicates) are shown next to the branches.

The tree (Figure 8), obtained from a dissimilarity matrix computed from allelic data of the 162 markers for each variety, gave a representation generally in accordance with the pedigree analysis (Table 6). The three genotypes which did not belong to the *H. brasiliensis* species (F4542, PUA8, PA31), were grouped together and were clearly distinct from most other *H. brasiliensis* genotypes, as reported by Feng et al. [22] using 87 EST-SSR markers. IRCA130, PB235 and PB260 genotypes, presenting a common ancestral parent (PB5/51), were well located on the same branch. IRCA109 and PB217 genotypes bred from the same ancestral parents were positioned on close branches. Even more clearly, CMB104 and CMB114, two full-sib genotypes, were localized at a quite a similar distance from their parents IRCA109 and PFB5. PR107 and its progeny PR255 were located on the same branch.

It is the first time that a large number of SNPs have been developed in *Hevea* after the publication by Pootakham et al [21] of 10 SNPs. The result indicates that these 172 SNPs would be useful for rubber tree genetics and breeding studies. Being heterozygous for at least one of the parents of recently published *Hevea* genetic maps, most of these SNPs could easily contribute in the near future to enhancing the density of these SSR-based genetic maps: 102 SNPs could be mapped in the PB260 x Fx3899 map [16], 103 SNPs in the PR255 x PB217 map [31], 100 SNP in the PB260 x Fx2784 map [32] and 78 SNPs in the PB260 x MDF180 map [33].

## Conclusions

This is the first *Hevea brasiliensis* transcriptome release covering the main tissues extracted from both clonal plant materials and derived hybrid plant materials obtained by open-pollination, and the first to investigate and analyze *Hevea brasiliensis* SNVs. The results of similarity identification, diversity of transcript localization, and variety of predicted functions from the 19,708 contigs obtained by our study, associated with the variety of tissues sampled demonstrate a cohesive approach to capturing the transcriptional landscape of whole rubber tree physiology.

Moreover, the public availability of the sequences, functional annotation and the global variant analysis, as well as the sequencing of raw data to be released from this study will provide a source of valuable information for biotechnology assays and genetic improvement of rubber trees, an addition to be used for a reference transcriptome for further sequencing projects.

As an allogamous plant with a recent history of selection, most of *Hevea* genotypes are highly heterozygous, opening the way for the development of a huge number of SNP markers.

## Methods

### Plant material

Tissues samples of *H. brasiliensis* from the RRIM600 cultivar, were collected at the E. Michelin Plantation in Itiquira (Mato Grosso state, Brazil) and at the Michelin Plantation in Ituberá (Bahia state, Brazil). Samples of male and female flowers, fruits, bark and latex from adult RRIM600 trees were collected at the E. Michelin Plantation and conserved in RNAlater (Life Technologies Carlsbad, CA, USA) until RNA extraction. Samples of stalks, petioles and leaves from grafted plants of RRIM600 and tissues (radicle, hypocotyl, epicotyl, albumen, cotyledon, leaves) from germinated seedlings of open-pollinated RRIM600 seeds were collected from a greenhouse at the Michelin Plantation in Ituberá. The tissue samples were stored in liquid nitrogen.

### Total RNA isolation and cDNA synthesis

Total RNA was isolated from 1 g of ground conserved tissue and extracted as described by Morcillo et al. (2006) [34]. RNA integrity was evaluated using a 2100 Bioanalyzer (Agilent Technologies, Santa Clara, CA, USA). Before mRNA purification, 18 RNA samples of RRIM600 tissues and 12 extracts of RRIM600 *OPS* (open-pollinated seedlings) were pooled. Poly(A) RNA was isolated from these two pools with oligo beads (dT) from the PolyATtract mRNA kit (Promega, Madison, WI, USA).

Following isolation, the mRNAs were fragmented using a 0.1 M zinc chloride solution in 0.1 M Tris-HCl pH7.0. Using these shorter fragments as templates, the first-strand cDNAs were synthesized using Roche random primers and the AMV reverse transcriptase from the cDNA Synthesis system and the GS Rapid Library kits (Roche Applied Science, Mannheim, Germany). Sequencing was carried out on a Roche/454 GS-FLX (Titanium) pyrosequencing platform.

### 454 sequencing and assembly of cDNA libraries

The cDNA libraries were amplified with emulsion PCR Lib-L (Roche Applied Science) and sequenced using the XLR70 sequencing kit and a 70 × 75 mm PicoTiterPlate (Roche Applied Science). Each library was sequenced in one region of the PicoTiterPlate.

All of the *H. brasiliensis* cDNA data were first filtered by quality scores, presence of adapters, PolyA/T tails and repetitive elements using preprocess.pl Perl script from est2Assembly [35], and then assembled into contigs using the Newbler *de novo* assembler algorithm of the gsassembler (Newbler version 2.7, Roche 454). Reads and assembled contigs were analyzed using R programming version 2.13.2 [36]. Scaffolding assembly was carried out by STM (Scaffolding using Translation Mapping) [09]. The STM method relies on the assumption that the gene set of the reference proteome, which will serve as a template for joining contigs into scaffolds, is sufficiently similar, and, in this way, all translated contigs matching a same reference protein can be assembled into a scaffold [09]. We used the *Ricinus communis* Protein sequence (amino acid translation) release 0.1 [37]

### Annotation, classification and comparison of assembled sequences

Contigs were compared to the set of proteome references by the BLAST algorithm (at an E-value threshold 10^-5^) against NCBI RefSeq, Plant Protein Database [38], *H. brasiliensis* assembled unique transcripts (PUT) of PlantGDB [39] and the complete NCBI nr database. We preferentially annotated contigs (with best BLAST hits) based first on similarity to Plant Protein RefSeq, then based on *H. brasiliensis* PUT nucleotides, and finally on Non-Redundant of nucleotides from the NCBI database. InterProScan version 4.8 was used for Gene Ontology and InterPro annotation [40] to connect *Hevea* transcript contigs with known gene ontology annotations. WEGO [41] software was used to perform GO annotation analysis and for plotting GO annotations. Also, attempting to phylogenetically classify the sequences, a BLAST using the Cluster of Orthologous Groups database was performed.

To gain an understanding of our set of transcripts mapping performed by GS Reference Mapper (Newbler version 2.7, Roche 454) against currently available *H. brasiliensis* sequences was carried out using the 2,311,497 reads generated by the Triwitayakorn et al. [13], obtained by sequencing *H. brasiliensis* shoot apex of the RRIM600 genotype, 30,094 *Hevea brasiliensis* EST sequences available in the NCBI database, including 9,860 ESTs (accession No EC600050–EC609910) from RRIM600 latex deposited in NCBI by Chow et al. [12].

For putative genes involved in the metabolic pathway, a KEGG annotation was performed using KAAS (KEGG Automatic Annotation Server) [42].

### Identification of Single Nucleotide Variants (SNVs) and Simple Sequence Repeat (SSR) loci

To detect nucleotide variants over the contigs, an alignment between reads and the contigs generated by Newbler as a reference was performed using the Burrows-Wheeler Aligner for long reads (BWA-SW) [43]. The results were used as input for the Bioconductor [44] Rsamtools version 1.6.0 package to obtain the possible nucleotide variants with a cut-off coverage of 4x, a threshold of 2 variants in the position and a base call quality cut-off of the PHRED score Q20 on average, where Q = -10 log10P, and the score stands for the probability of a wrong base being called.

To check wether or not SNV was responsible for a non-synonym alteration in the amino acid composition from the contig, a gene prediction analysis was carried out by the GlimmerHMM Eukaryotic Gene-Finding System [45] using the *Arabidopsis thaliana* training model in an attempt to identify the Protein Coding Sequence, for correct verification of the SNV substitution type made by the R/Bioconductor script.

Searches for SSRs from the contig data set were performed by Microsatellite Identification Tool (MISA) version 1.0 [46]. The definition of microsatellites (unit size/ minimum number of repeats) was set as mononucleotides repeats if the same nucleotide was repeated at least 10 times (1/10), di (2/6), tri (3/5), tetra (4/5), penta (5/5), or hexanucleotides (6/5), and 100 as the maximum number of bases interrupting two SSRs in a compound microsatellite (microsatellites consisted of more than a single repeat type).

### Protein characterization

The molecular weight and the isoelectric point analyses for proteins were performed by EMBOSS version 6.4.0 [47] and the generated output analyzed by R programming language.

### Development of SNP markers

The 191 detected SNPs were validated using KASP genotyping chemistry (KBioscience Ltd., Hoddesdon, UK) on 23 *Hevea* genotypes including different species (*H. brasiliensis, benthamiana* and *pauciflora*) and related genotypes (Table 6). Rubber tree total genomic DNA was extracted from fresh leaves following a previously described method [48]. For each SNV position, three primers were designed in a region of +50 and -50 pb around the nucleotide variation by KBioscience with PrimerPicker [49]. The 191 *Assay Mixes* contained two allele-specific forward primers (12 μM) able to anchor specifically to the 3’ position in the nucleotide variant, and a reverse primer (30 μM). Genotyping was performed using the traditional KASP genotyping chemistry on a LightCycler 480 II (Roche) using a 384-well plate. The PCR reactions were carried out in 4 μL containing 2 ng of genomic DNA, 2 μL Master MIX 2x, 2.2 mM MgCl_2_ and 0.055 μL *Assay Mix*. One denaturation cycle was performed at 95°C for 15 min, prior to 10 denaturation cycles at 94°C for 20 s, annealing at 65°C for 1 min (- 0.8°C/cycle), followed by 40 denaturation cycles for 20 s, annealing at 57°C for 1 min. The end point fluorescence signal was measured and plotted on two axes. All genotype calls were manually checked and ambiguous data points that failed to cluster were scored as missing data. Each nucleotide variant was scored as allelic data (A = 1, C = 2, G = 3, T = 4, not determined data =0). These data were used to calculate a genetic dissimilarity matrix using the simple matching dissimilarity index (d_ij_) between pairs of accessions (units) [50].

$$d_{\mathrm{ij}}=1-\frac{1}{L}\overset{L}{\underset{l=1}{\sum }}\frac{m_{l}}{2}$$

where d_ij_ represents the dissimilarity between units i and j, L represents the number of loci, and m_l_ represents the number of matching alleles between i and j for locus l. From the dissimilarity matrix, a Neighbor-Joining tree [51] was computed using the DARwin software version 5.0.158 (Dissimilarity Analysis and Representation for Windows, http://darwin.cirad.fr/darwin[50]. Branch robustness was tested using 1000 bootstraps.

## Competing interests

The authors declare that they have no competing interests.

## Authors’ contributions

LRS and DGP worked on assembly, annotation, pathway analysis, screening for molecular markers. LRS and DG wrote the manuscript. DMK and DG generated the RNA libraries. DG, MFN, LGPA, VRR, MG, and ALG performed the raw data generation. RR, VL and DG developed the SNPs. AF, JCMC, ATRV, WASJ, LLC and DG designed the study. All the authors read and approved the final manuscript.

## Supplementary Material

Additional file 1: Table S1 Characteristics of SNP loci developed in *Hevea*. The 454-data generated can be accessed and downloaded at http://scarecrow.fmrp.usp.br/heveabr/. Click here for file
